# Hospice Care Preferences and Its Associated Factors among Community-Dwelling Residents in China

**DOI:** 10.3390/ijerph19159197

**Published:** 2022-07-27

**Authors:** Huijing Lin, Eunjeong Ko, Bei Wu, Ping Ni

**Affiliations:** 1 School of Nursing, Tongji Medical College, Huazhong University of Science and Technology, Wuhan 430030, China; linhuijing@hust.edu.cn; 2 School of Social Work, San Diego State University, San Diego, CA 92182, USA; eko@sdsu.edu; 3 Rory Meyers College of Nursing, New York University, New York, NY 10010, USA; bei.wu@nyu.edu; 4 NYU Aging Incubator, New York University, New York, NY 10010, USA

**Keywords:** hospice care, residents, life-sustaining treatment, end-of-life care, awareness, medical insurance

## Abstract

Hospice care is a comprehensive approach addressing patients’ physical, psychosocial, and spiritual needs at the end of life (EoL). Despite the recognition of its effectiveness in improving the quality of EoL care, little is known about hospice care in mainland China. In this study, we aimed to examine the preferences for hospice care and its related factors among community-dwelling residents in mainland China. Participants were recruited using a convenience sampling method, and 992 community-dwelling residents responded to an online survey from June 2018 to August 2019. The majority (66.7%) of the participants were female, and the mean age was 48.4 years. Approximately 28% of the participants had heard of hospice care, and 91.2% preferred to receive hospice care if diagnosed with a terminal illness. Participants who had heard of hospice care, and with higher levels of education (bachelor’s degree or above) and health insurance coverage were more likely to accept hospice care than their counterparts. Community-based education on hospice care is imperative to improve public knowledge and the acceptance of hospice care. Meanwhile, there is a need to develop policies to integrate and expand hospice care into clinical settings.

## 1. Introduction

China is a country with a large population of 1.44 billion by 2020, ranking first in the world [1]. Innovations in technology have made significant contributions to longevity [2]. However, increasing life expectancy and an accelerated aging population have brought much attention to the quality end-of-life (EoL) care globally, including in China [3]. Hospice care, a comprehensive approach, serves to meet the physical, psychosocial, and spiritual needs of individuals with a terminal illness and their family members [4]. Strong evidence shows that hospice care improves the quality EoL care [5,6,7], reduces medical costs [3,8,9], meets people’s goals for comfortable care, and limits burdensome therapies [10,11].

Hospice care in China is relatively nascent compared to some Western countries (e.g., United States, Canada, England, Australia), yet growing steadily [12,13,14]. Since hospice care was introduced in 1988 [15], it has made great progress, and by 2017, there were 2343 medical institutions with hospice care departments in China [16]. Nevertheless, hospice care facilities in mainland China are significantly limited as compared with Hong Kong [17] and Taiwan, China [18]. Hospice care in China is provided in three forms: inpatient hospice care, outpatient hospice care, and home care [19]. However, at present, hospice care in mainland China is mostly provided in hospitals where curative treatments are still the mainstream care in the health care system, and hospice wards are in great shortage [19]. A recent report indicated that there were just 510 (1.4%) hospitals with hospice departments by 2020 in mainland China [20].

The utilization rate of hospice care in China is far below that of developed countries in North America [21,22], Europe [23,24], and East Asia (such as Japan) [25]. A recent study showed that only 10% of dying cancer patients receive hospice care annually [26]. Previous studies have identified that knowledge of hospice care [27,28], the experience of a loved one’s death [29], the experience of caring for a loved one with chronic diseases [29], and several individual characteristics, such as age [17,22], gender [22,30], and educational levels [30] were significantly associated with hospice care preference.

In China, the public’s attitude toward hospice care is generally positive. A study conducted in Hong Kong found that about 87.6% of adults preferred to receive hospice care (i.e., comfort care) rather than life-sustaining treatment (LST) when they had a terminal illness [17]. A recent study in mainland China found that most patients or their families (90.7%) had positive attitudes toward hospice care for their terminal conditions [27]. However, the public’s awareness of hospice care is low in China, compared with that in developed countries with advanced hospice care systems [28,31,32]. For example, a recent study with community residents in Hangzhou, China, found that only 50.3% of adult populations aged 40 and above in China were aware of hospice care [28], as compared to 86% and 76% of the general population in the United States [33] and Northern Ireland [34], respectively. Awareness of hospice care is even lower among cancer patients (5.8%) [14] and oncologists in China [35]. Hospice care is often perceived as appropriate only for those facing imminent death in China. Thus, receiving hospice care is giving up hope [27].

In response to the Healthy China 2030 Action Plan and the Ten-year Health-care Reform Project, the Chinese government has made efforts to promote hospice care, including launching national hospice pilot projects in various regions in mainland China [36]. Despite heightened public interest in quality EoL care, mainland China ranked 71st among 80 countries and regions in quality of death according to the 2015 Quality of Death Index, published by the Economist Intelligence Unit [37]. This reflects limited accessibility to hospice care and a lack of national policies and guidelines as well as standardized hospice practice, which negatively impact quality EoL care in China [37].

With the government’s initiatives for hospice care, understanding the general public’s awareness of hospice care, hospice care preferences, and associated factors can further promote the development of policies and the legitimation support for hospice care [28]. Meanwhile, the public’s attitude toward hospice care can impact willingness to accept the care [38] and affect EoL decisions [39]. Therefore, it is important to understand the adult population’s views toward hospice care. While several studies on this topic in China have broadened our understanding of knowledge and attitudes toward hospice care, most have focused primarily on the perspectives of health care providers [35,40] and patients [27,41]. To our knowledge, there is only one hospice study conducted with the general population in Hangzhou [28], which found that 42% of the participants had basic knowledge of hospice care, and participants with a better understanding level and who lived in urban areas preferred hospice care, but this study focused on adults aged 40 and above with a relatively small sample size (*n* = 519). There is a lack of community-based data regarding awareness of hospice care and factors affecting preferences toward hospice among adults aged 18 and older. Thus, with this study, we sought to examine the general adult population’s preferences for hospice care and identify its associated factors in mainland China.

## 2. Materials and Methods

### 2.1. Study Design and Sample

This was a cross-sectional design that surveyed community-dwelling residents in Wuhan, China. An online survey was conducted via a commonly used survey platform using a convenience sampling method. Ethical approval of the research protocol was granted by the Institutional Review Board of Huazhong University of Science and Technology, and signed informed consent was obtained from each participant when they clicked the button “Yes, I am informed and volunteered to participate in the study” on the first page of the online survey. Data were collected from June 2018 to August 2019.

Participants were recruited from 8 of 13 communities in Wuhan, China, using a convenience sampling method. The research team members contacted the administrators and staff of the community districts and explained the purpose and procedures of the study. Upon receiving approval to proceed with the study from the staff, a notice with a description of the study and the Quick Response (QR) code was posted on the community bulletin boards, which is used to post public messages via post notices, leaflets, and advertisements. Those interested in participating in the study could scan the QR code on the notice to access the link for the online survey. Eligibility criteria for study participation included: community-dwelling residents aged 18 and older, Chinese, and living in Wuhan. In total, 1114 participants expressed interest and completed the survey; however, 122 were excluded as they did not meet the eligibility criteria (12 were excluded by age, 110 were excluded by residency). Finally, a total of 992 participants were included in the study.

### 2.2. Data Collection

Participants who were interested in this study could scan the QR code on the notice to access the online link for the survey. After opening the survey link, participants were directed to review and follow the informed consent procedures. Protection of participants’ anonymity and confidentiality were ensured. To make sure that there were no duplicate participants responding to the study, we limited the participant ability to open the link using their mobile device to one time.

### 2.3. Measures

Participants’ awareness of and preference for hospice care, and their socio-demographic information were measured based on parts of an existing tool designed by Chung and colleagues [17], which was used to explore the knowledge, attitudes, and preferences of advance directives, EoL care, and place of care and death among adults in China.

Awareness of hospice care was assessed by asking the participants, “Have you ever heard of hospice care?” Response categories were “yes” and “no”. For those who answered “no”, a brief explanation of hospice care was provided. Hospice care is defined as a patient-centered care which aims at improving quality EoL through pain and symptom management, but cannot prolong life [42,43].

Preference for hospice care was assessed by asking the participants “If you were diagnosed with a terminal illness, would you like to accept hospice care?” The response categories were briefly “yes” and “no”.

Awareness of and preference for LST. Awareness of LST was assessed by asking participants, “Have you ever heard of LST, an active medical therapy, which has the potential to postpone patients’ death, but cannot recover health and would bring pain or discomfort?” [44,45], with response categories of “yes” or “no”. For those who answered “no”, an introduction of types of LST (mechanical ventilation, cardiac monitoring, cardiopulmonary resuscitation, feeding tube, blood transfusion, etc. [44,45]) was provided. Preference for LST was measured by asking, “If you were in a severe condition in which LST (e.g., mechanical ventilation, feeding tube) could not recover your health, and you might even have to rely on it over long-term to prolong your life, would you like to accept it?” Response categories were “yes” or “no”. Then, reasons for accepting or rejecting LST were provided in the following questions in an open-ended format.

Socio-demographic information included gender, age, educational levels, religious belief, marital status, type of housing (e.g., public apartment or private housing), occupation, financial status (not adequate, somewhat adequate, adequate), type of health insurance (free medical service, commercial insurance, and others), the breadwinner of the family (self, spouse, and others), and having a government subsidy (yes, no/not clear). Health-related information included: (1) self-rated health (good, fair, poor), (2) having chronic diseases (yes, no, not clear), (3) having a terminal illness (yes, no, not clear), (4) experience with caring for family members with chronic diseases (no family member with chronic diseases, I was/am not the caregiver, and I was/am the caregiver) and (5) experience with caring for family members with a terminal illness (no family member with a terminal illness, I was/am not the caregiver, and I was/am the caregiver).

### 2.4. Data Analysis

Data analysis was performed using the Statistical Package for Social Science (SPSS), version 24.0 [46]. Descriptive statistics were calculated to present participants’ socio-demographic, health-related information, awareness of and preference for hospice care and LST, and reasons for accepting/rejecting LST. Group differences in sample characteristics were compared between those who accepted hospice care and those who refused hospice care through bivariate analysis using chi square tests (χ^2^). Then, binary logistic regression analysis was conducted to identify the associated factors related to hospice care preference. Participants’ socio-demographic, health-related information, and awareness of hospice care and LST were independent variables. Hospice care preference was the dependent variable. *p* < 0.05 was statistically significant. An odds ratio (OR) with 95% confidence interval (CI) was estimated for each outcome factor.

## 3. Results

### 3.1. Characteristics of the Participants

Participants’ socio-demographics and health-related information are presented in Table 1. Two-thirds of the participants (66.7%) were female with an average age of 48.4 (SD = 12.2). About two-thirds (67.9%) had a bachelor’s degree or above. The majority were living in private apartment (81.6%) and 44.7% reported their financial status as somewhat adequate. In terms of health status, half of participants (54.0%) rated their health as fair, 251 (25.3%) had chronic diseases, and 16 (1.6%) had a terminal illness. About 22.6% were caregivers of family members with chronic diseases, and 6.8% reported caring for family members with a terminal illness.

### 3.2. Awareness of and Preference for Hospice Care and LST

Only 28.1% of the participants reported to be aware of hospice care. When the concept of hospice care was introduced, the majority (91.2%) preferred to use hospice care if they were diagnosed with a terminal illness. In terms of LST-related information, about 62.8% of the participants reported to have heard of LST, and most participants (88.9%) were not willing to prolong life by relying on LST. The most commonly cited reason for rejecting LST was “to avoid burdening the family”, and the most common reason for accepting LST was that they “cannot bear to leave my family” (see Table 2).

### 3.3. Factors Related to Hospice Care Preference

In Table 3, bivariate analysis results suggest that participants who accepted hospice care and those who rejected hospice care had significant differences in their age, gender, educational level, religious belief, marital status, financial status, health insurance status, government subsidy status, self-rated health status, terminal illness status, and awareness of hospice care and LST. In the binary logistic regression model, having heard of hospice care before (OR = 5.837, *p* < 0.001), with the educational level of bachelor’s degree or above (OR = 1.825, *p* < 0.001) and having health insurance (OR = 1.610, *p* = 0.001) were significantly associated with hospice care preference (Table 4). Participants who had heard of hospice care prior to the study were 5.84 times more likely to accept hospice care than those who had not. Similarly, participants who had a bachelor’s degree or above and had health insurance were 1.83 and 1.61 times more likely, respectively, to prefer hospice care as compared to those with only secondary school education and below, and no health insurance.

## 4. Discussion

### 4.1. Preference for Hospice Care among Community-Dwelling Residents

The results of our study show that the majority (91.2%) of participants indicated that they would accept hospice care if diagnosed with a terminal illness, while only 8.8% preferred active LST to prolong their life even if it brought pain and discomfort. This lower prevalence of willingness to use LST and higher preference for hospice care was similar to findings from previous studies [28,47]. Participants may view relying on artificial measures as futile when they cannot cure diseases [47] and rather prefer hospice care which can satisfy needs for relieving pain and providing comfort [3,9]. This may explain why participants tend to face death rationally and avoid unnecessary prolonging of life when suffering an irreversible coma or persistent vegetative state [47]. Thus, effective measures, such as policy support and public education, should be taken to improve the hospice availability.

### 4.2. Associated Factors Related to Hospice Care Preference among Community-Dwelling Residents

Consistent with previous studies [27,28], participants who had heard of hospice care were more likely to accept hospice care. Participants who had heard of hospice care may be more knowledgeable about the program and services; therefore, they were more likely to accept hospice care. It needs noting that, in our study, only 28.1% of respondents heard of hospice care, which is much lower than the result of another study in China, where 50.3% of community residents were aware of hospice care [28]. The lack of awareness of hospice care in Mainland China might be contributed to by the lack of systematic policy support, limited public educational campaigns, and lack of comprehensive academic and practical curriculums and training on hospice care [27,28,48]. Promoting public awareness on hospice care would require taking active steps to provide education. Ni and colleagues [27] found that the use of media such as Internet or television, newspapers or magazines, as well as education by health care providers were effective approaches to enhance knowledge about hospice care. There is a need to promote public knowledge about hospice care by facilitating public health educational forums or workshops in collaboration with community health care centers [15]. In addition, strengthening medical curriculum on hospice care [27,35] and developing hospice care programs in hospitals [15] can increase the opportunities for physicians, nurses, patients, and their family members to improve awareness and increase utilization of hospice care services. In particular, the misperception of hospice care as giving up hope for life might evoke strong emotions against cultural values of family-centered care in China. Hence, providing more information on the goals of hospice, its scope of practice, and benefits may help address these misperceptions [49].

Participants with higher levels of education were more likely to prefer hospice care. A previous study investigating the relationship between educational attainment and health care access and use found that people with higher levels of education were more likely to be insured [50], which was also a significant predictor of accepting hospice care in our study. Moreover, participants with higher educational levels may have a greater knowledge about the cost-effectiveness of hospice [51,52], as well as other benefits and limitations [33]; thus, they were more likely to be receptive toward hospice care. With the existence of the misinterpretation of hospice care [53], participants with lower levels of education may be less able to distinguish the difference between quality and length of life; thus, they may try to prolong life to achieve the goal of “good” quality of life. It is important for health care staff to assess individuals’ perceptions on quality EoL and provide education on the purpose and value of hospice care.

Participants who had health insurance were more likely to accept hospice care than those who did not have health insurance. Costs of care at the EoL stage account for a large proportion of all expenses, especially in the last month of life [9]. In countries with high quality of death (e.g., Japan, Britain, Singapore), hospice care fees are covered by universal health insurance [28]. However, fees for hospice care only partially covered by health insurance reimbursement in mainland China [54], leading to economic burdens on the patients and family. Those who do not have health insurance may receive free care if they can obtain philanthropic support [15] or pay out-of-pocket, which is difficult and rare. In our study, only 67.8% of participants had health insurance, which may influence their decision about hospice care. Of note, hospice care paid per day has been officially implemented from 1 January 2022 in Sichuan, China, which suggests that fees for hospice care per day for residents in tertiary, secondary, primary, and below medical institutions are 350 yuan (equivalent to 55.11 USD), 300 yuan (equivalent to 47.23 USD), and 260 yuan (equivalent to 40.94 USD), respectively, of which about 20–30% should be paid out-of-pocket for those with health insurance [55,56]. Xie and colleagues [28] found that Chinese community residents wished for out-of-pocket costs for hospice care of less than 200 yuan (equivalent to 31.49 USD) per day. Many hospice services are not reimbursed by the national health insurance in China [54]. Policy makers should be aware of the fact that hospice care is indeed a highly sustainable and cost-effective program, because it improves quality care at EoL while reducing expensive costs of invasive LST measures [51,52]. This indicates the importance of government support for wide access to hospice care as well as establishing a health insurance system that covers the full cost of hospice care. Efforts are also needed to improve hospice facilities for better hospice services.

### 4.3. Strengths and Limitations of the Study

This study is one of the few studies exploring the awareness of and attitudes toward hospice care among community-dwelling residents in mainland China. While this study yielded valuable information that fills knowledge gaps on this topic, there are several limitations. First, we used a convenient sampling method that included residents of eight communities in Wuhan city, which may limit the generalizability of the study findings. In addition, the online survey method may exclude those with limited access to computers or who lack digital literacy (e.g., older adults with low socioeconomic status). Second, environmental factors, such as access to health care and community-level socioeconomic status, were not investigated, which may affect hospice care preferences. Third, our study did not include family-related variables (e.g., relationship to the family, perceived burden on family), which may impact preferences for hospice care. Future studies including family members or measures assessing family relationships or attitudes toward hospice care would be beneficial to better assess hospice care preference.

## 5. Conclusions

In our study, community-dwelling residents had a relatively low level of awareness of hospice care but more readily engaged in hospice care services, which indicated they value high-quality EoL care. It is important to strengthen hospice education in order to improve public awareness and acceptance of hospice care, thereby improving quality EoL care. To enhance public education on hospice care, the systematic development and implementation of culturally appropriate educational programs are critical. When discussing EoL care, health care providers need to assess both the patient’s and family’s knowledge of hospice care and provide the necessary education accordingly. Collaborating with community-based health organizations along with the use of media, developing community-based education, and collaborating with community stakeholders can be effective approaches for promoting hospice education. In addition, the establishment of relevant regulations and policies for hospice care in mainland China is essential to expand hospice care practice.

## Figures and Tables

**Table 1:** Characteristics of participants (*n* = 992).

Variables	Category	*n* (%)
Age (years) *	18–29	55 (5.5)
	30–39	197 (19.9)
	40–49	316 (31.9)
	50–59	231 (23.3)
	60+	193 (19.4)
Gender	Male	330 (33.3)
	Female	662 (66.7)
Educational level	Secondary school education and below	318 (32.1)
	Bachelor’s degree or above	674 (67.9)
Religious beliefs	None	896 (90.3)
	Buddhism	72 (7.3)
	Christianity	12 (1.2)
	Others	12 (1.2)
Marital status	Married/cohabited	853 (86.0)
	Unmarried/divorced/separated/widowed	139 (14.0)
Type of housing	Public apartment	81 (8.2)
	Private housing	809 (81.6)
	Others	102 (10.2)
Occupation	Student	3 (0.3)
	Medical-related occupation	69 (7.0)
	Non-medical related occupation	920 (92.7)
Financial status	Not adequate	160 (16.1)
	Somewhat adequate	443 (44.7)
	Adequate	389 (39.2)
Health insurance	Insured	673(67.8)
	Uninsured	319 (32.2)
Type of health insurance ^a^	Free medical service	303 (45.0)
	Commercial insurance	251 (37.3)
	Others	119 (17.7)
Have a government subsidy	No/not clear	765 (77.1)
	Yes	227 (22.9)
Breadwinner of the family	Self	465 (46.9)
	Spouse	411 (41.4)
	Others (sons/daughters, parents, government)	116 (11.7)
Self-rated health	Good	388 (39.1)
	Fair	536 (54.0)
	Poor	68 (6.9)
Have chronic diseases	Yes	251 (25.3)
	No	555 (55.9)
	Not clear	186 (18.8)
Have a terminal illness	Yes	16 (1.6)
	No	874 (88.1)
	Not clear	102 (10.3)
Experience caring for family members with chronic diseases	No family member with chronic diseases	527 (53.1)
	I was/am not the caregiver	241 (24.3)
	I was/am the caregiver	224 (22.6)
Experience caring for family members with a terminal illness	No family member with a terminal illness	847 (85.4)
	I was/am not the caregiver	77 (7.8)
	I was/am the caregiver	68 (6.8)

* Mean: 48.4, standard deviation: 12.2, range 20–86 years old. ^a^ *n* = 673 for only those who had health insurance.

**Table 2:** Hospice care and LST awareness and preferences among participants (*n* = 992).

Variables	Category	*n* (%)
Heard of hospice care	Yes	279 (28.1)
	No	713 (71.9)
If you were diagnosed with a terminal illness, would you like to accept hospice care?	Yes, I want hospice care to give me comfort even if it cannot prolong my life	905 (91.2)
	No, I want active LST to prolong my life as much as possible even it brings pain and discomfort	87 (8.8)
Heard of LST, an active medical therapy, which has the potential to postpone patients’ death, but cannot recover health and would bring pain or discomfort	Yes	623 (62.8)
	No	369 (37.2)
If you were in a severe condition in which LST (e.g., mechanical ventilation, feeding tube) could not recover your health, and you might even have to rely on it in the long term to prolong your life, would you like to accept it?	Yes	110 (11.1)
	No	882 (88.9)
Reasons for accepting LST (may choose more than one answer) *	I couldn’t bear to leave my family	74 (67.3)
	Try if there is a chance	38 (34.5)
	I want to live longer	33 (30.0)
	Others	10 (9.2)
Reasons for rejecting LST (may choose more than one answer) ^a^	To avoid of burdening the family	628 (71.2)
	The treatments are too painful	568 (64.4)
	The effects of treatments are too limited	480 (54.4)
	Too many sequelae from treatments	218 (24.7)
	I’ve lived long enough	36 (4.1)
	Others	56 (6.2)

* *n* = 110 for only those who accepted LST, ^a^ *n* = 882 for only those who rejected LST. Abbreviation: LST, life-sustaining treatment.

**Table 3:** Chi-square test identifying factors related to hospice care preference (*n* = 922).

Variables	Category	Hospice Care Preference		Total (%) (*n* = 992)	χ^2^	*p*
		Accept (%) (*n* = 905)	Reject (%) (*n* = 87)			
Age	18–29	48 (87.3)	7 (12.7)	55 (5.5)	12.683	0.013 *
	30–39	178 (90.4)	19 (9.6)	197 (19.9)		
	40–49	279 (88.3)	37 (11.7)	316 (31.9)		
	50–59	213 (92.2)	18 (7.8)	231 (23.3)		
	60+	187 (96.9)	6 (3.1)	193 (19.4)		
Gender	Male	311 (94.2)	19 (5.8)	330 (33.3)	5.609	0.011 *
	Female	594 (89.7)	68 (10.3)	662 (66.7)		
Educational level	Secondary school education and below	294 (92.5)	24 (7.5)	318 (32.1)	14.873	<0.001 *
	Bachelor’s degree or above	611 (90.7)	63 (9.3)	674 (67.9)		
Religious belief	None	817 (91.2)	79 (8.8)	896 (90.3)	29.883	<0.001 *
	Buddhism	70 (97.2)	2 (2.8)	72 (7.3)		
	Christianity	6 (50.0)	6 (50.0)	12 (1.2)		
	Others	12 (100.0)	0 (0)	12 (1.2)		
Marital status	Married/cohabited	773 (90.6)	80 (9.4)	853 (86.0)	4.817	0.023 *
	Unmarried/divorced/separated/widowed	132 (95.0)	7 (5.0)	139 (14.0)		
Type of housing	Public apartment	78 (96.3)	3 (3.7)	81 (8.2)	8.563	0.306
	Private housing	728 (90.0)	81 (10.0)	809 (81.6)		
	Others	99 (97.1)	3 (2.9)	102 (10.2)		
Occupation	Student	3 (100)	0 (0)	3 (0.3)	0.458	0.795
	Medical-related occupation	62 (89.9)	7 (10.1)	69 (7.0)		
	Non-medical related occupation	840 (91.3)	80 (8.7)	920 (92.7)		
Financial status	Not adequate	155 (96.9)	5 (3.1)	160 (16.1)	8.909	0.012 *
	Somewhat adequate	404 (91.2)	39 (8.8)	443 (44.7)		
	Adequate	346 (88.9)	43 (11.1)	389 (39.2)		
Health insurance	Insured	624 (92.7)	49 (7.3)	673(67.8)	5.802	0.016 *
	Uninsured	281 (88.1)	38 (11.9)	319 (32.2)		
Type of health insurance ^a^	Free medical service	282 (93.1)	21 (6.9)	303 (45.0)	4.293	0.232
	Commercial insurance	233 (92.8)	18 (7.2)	251 (37.3)		
	Others	109 (91.6)	10 (8.4)	119 (17.7)		
Have a government subsidy	No/not clear	687 (89.8)	78 (10.2)	765 (77.1)	21.998	0.003 *
	Yes	218 (96.0)	9 (4.0)	227 (22.9)		
Breadwinner of the family	Self	426 (91.6)	39 (8.4)	465 (46.9)	4.440	0.109
	Spouse	368 (89.5)	43 (10.5)	411 (41.4)		
	Others (sons/daughters, parents, government)	111 (95.7)	5 (4.3)	116 (11.7)		
Self-rated health	Good	352 (90.7)	36 (9.3)	388 (39.1)	11.117	0.004 *
	Fair	498 (92.9)	38 (7.1)	536 (54.0)		
	Poor	55 (80.9)	13 (19.1)	68 (6.9)		
Have chronic diseases	Yes	235 (93.6)	16 (6.4)	251 (25.3)	3.316	0.190
	No	505 (91.0)	50 (9.0)	555 (55.9)		
	Not clear	165 (88.7)	21 (11.3)	186 (18.8)		
Have a terminal illness	Yes	10 (62.5)	6 (37.5)	16 (1.6)	18.420	<0.001 *
	No	798 (91.3)	76 (8.7)	874 (88.1)		
	Not clear	97 (95.1)	5 (4.9)	102 (10.3)		
Experience caring for family members with chronic diseases	No family member with chronic diseases	474 (89.9)	53 (10.1)	527 (53.1)	5.441	0.066
	I was/am not the caregiver	218 (90.5)	23 (9.5)	241 (24.3)		
	I was/am the caregiver	213 (95.1)	11 (4.9)	224 (22.6)		
Experience caring for family members with a terminal illness	No family member with a terminal illness	770 (90.9)	77 (9.1)	847 (85.4)	0.910	0.635
	I was/am not the caregiver	71 (92.2)	6 (7.8)	77 (7.8)		
	I was/am the caregiver	64 (94.1)	4 (5.9)	68 (6.8)		
Heard of hospice care	Yes	237 (84.9)	42 (15.1)	279 (28.1)	112.076	<0.001 *
	No	343 (48.1)	370 (51.9)	713 (71.9)		
Heard of LST, an active medical therapy, which has the potential to postpone patients’ death, but cannot recover health and would bring pain or discomfort	Yes	580 (93.1)	43 (6.9)	623 (62.8)	7.305	0.005 *
	No	325 (88.1)	44 (11.9)	369 (37.2)		

* *p* < 0.05. ^a^ *n* = 673 for only those who had health insurance. Abbreviation: LST, life-sustaining treatment.

**Table 4:** Binary logistic regression model identifying factors related to hospice care preference (*n* = 922).

Independent Predictors (Reject vs. Accept)	OR	95% CI	*p*	*β* (SE)
Heard of hospice care				
No (reference)	1			
Yes	5.837	4.057–8.398	<0.001 *	1.764
Educational level				
Secondary school education and below (reference)	1			
Bachelor’s degree or above	1.825	1.364–2.442	<0.001 *	0.602
Health insurance				
Uninsured (reference)	1			
Insured	1.610	1.204–2.153	0.001 *	0.476

* *p* < 0.05. Abbreviations: OR, odds ratio; CI, confidence interval; SE, standard error.

## Data Availability

Data may be provided by the corresponding author upon reasonable request.
